# Postpartum Depression and Anxiety among Lebanese Women: Correlates and Scales Psychometric Properties

**DOI:** 10.3390/healthcare11020201

**Published:** 2023-01-09

**Authors:** Eva Hobeika, Diana Malaeb, Sahar Obeid, Pascale Salameh, Elie Hobeika, Miguella Outayek, Marwan Akel, Nelly Kheir, Zaki Sleiman, Habib Barakat, Souheil Hallit

**Affiliations:** 1 Faculty of Arts and Sciences, Holy Spirit University of Kaslik, Jounieh P.O. Box 446, Lebanon; 2 College of Pharmacy, Gulf Medical University, Ajman P.O. Box 4184, United Arab Emirates; 3 Department of Social and Education Sciences, School of Arts and Sciences, Lebanese American University, Jbeil 4504, Lebanon; 4 School of Medicine, Lebanese American University, Byblos 4504, Lebanon; 5 INSPECT-LB: Institut National de Sante Publique, Epidemiologie Clinique et Toxicologie-Liban, Beirut 1103, Lebanon; 6 Department of Primary Care and Population Health, University of Nicosia Medical School, Nicosia 2408, Cyprus; 7 Faculty of Pharmacy, Lebanese University, Beirut 1103, Lebanon; 8 Clinical Obstetrics and Gynecology, American University of Beirut Medical Center, Beirut 4505, Lebanon; 9 School of Pharmacy, Lebanese International University, Beirut 1105, Lebanon; 10 Research Department, Psychiatric Hospital of the Cross, Jal Eddib P.O. Box 198, Lebanon; 11 Rizk Hospital, Lebanese American University, Beirut 4505, Lebanon; 12 Obstetrics and Gynecology Department, Notre Dame des Secours University Hospital Center, Street 93, Byblos Postal Code 3, Lebanon; 13 School of Medicine and Medical Sciences, Holy Spirit University of Kaslik, Jounieh P.O. Box 446, Lebanon; 14 Applied Science Research Center, Applied Science Private University, Amman 11931, Jordan

**Keywords:** postpartum, depression, anxiety, factors, Lebanon

## Abstract

**Background**: We found that it was important to fill a gap in the literature and check the psychometric properties of the Edinburgh Postnatal Depression Scale (EPDS) and Perinatal Anxiety Screening Scale (PASS) in the Arabic language and delineate factors associated with postnatal depression (PPD) and anxiety (PPA) among Lebanese women 4–6 weeks after delivery. **Methods**: This cross-sectional study carried out between July 2018 and March 2019 enrolled 295 participants who came for a postnatal checkup at four clinics. **Results**: The EPDS and PASS scales’ items converged over two- and four-factor solutions, explaining 62.51% and 53.33% of the variance, respectively (KMO EPDS = 0.816, αCronbach EPDS = 0.826; KMO PASS = 0.878, αCronbach PASS = 0.920; Bartlett’s test of sphericity *p* < 0.001). Higher postpartum anxiety (Beta = 0.256), higher postpartum insomnia (Beta = 0.079), having hypotension during pregnancy (Beta = 2.760), and having a second (Beta = 1.663) or a third baby or more (Beta = 2.470) compared with the first one were significantly associated with higher postpartum depression. Higher postpartum depression (Beta = 1.33) was significantly associated with higher postpartum anxiety, whereas having a baby through a planned pregnancy (Beta = −4.365) and having a baby who ate regularly (Beta = −3.639) were significantly associated with lower postpartum anxiety. **Conclusion**: Depression and anxiety prevalence rates in the Lebanese population were higher compared with other countries, which may be due in part to the differences in regional, social and environmental culture.

## 1. Introduction

Childbirth causes important changes in the mother’s physiology, inducing an onset of psychopathological symptoms that differ in intensity and frequency [1,2]. These psychopathological symptoms include various forms of anxiety and depression, surfacing especially in the period when the mother is recovering physically from giving birth. In addition, short- and long-term impacts on the mother’s and child’s well-being can be detected [3]. Baby blues is a short-lasting condition primed by childbirth, featuring mild symptoms such as anxiety, mood swings and amplified emotional reactivity, with a minimal impact on functioning [4]. Baby blues are reported in 15–85% of women within the first 10 days after birth, with a peak incidence on the fifth day [4]. Although the baby blues postpartum occurrence is common, transient and does not require intervention, the relevance of its recognition should be noted as it is postulated as a risk factor for subsequent postpartum depression [5]. The most statistically and clinically relevant psychological complication related to giving birth to children is postpartum depression. About 10–15% of women giving birth may develop postpartum depression, with differences between population groups and geographical locations [6].

The Diagnostic and Statistical Manual of Mental Disorders, 5th Edition (DSM-5), states that the “Postpartum Onset” (a class of major depressive disorders) is characterized by symptoms appearing 4–6 weeks after delivery. More specifically, postpartum depression symptoms include disturbances in appetite and sleep, energy loss, the sensation of guilt, diminished attentiveness and plausible suicidal thoughts [7]. Recent findings showed a prevalence rate of 19.8% of postpartum depression after birth (between 19.5 and 20.0%) [8].

A better understanding of postpartum depression first requires the delineation of factors that influence its occurrence rate. According to population-based studies, the following risk factors of postpartum depression are significant (in the prenatal, perinatal and postnatal phases): (a) patient history of depression [9,10,11]; (b) psycho-social factors (such as marital problems, decreased social support and a negative life) [10,12]; (c) socio-economic factors (such as unemployment, poverty and a low education level) [9,10,11,12]; (d) medical factors, including complications in delivery and pregnancy [9,11], substance (alcohol/nicotine) abuse while pregnant [9], poor sleep [10,12], post-delivery wound discomfort and drastic maternity blues [10]; and (e) factors related to the child (such as the sex of the baby, difficulties in breastfeeding and an undesired pregnancy) [9].

To screen for postpartum depression, the Edinburgh postnatal depression scale (EPDS) is a well-accepted tool and was used in the majority of studies. Several versions of this scale in different languages have been created and validated in several countries, such as Brazil [13], Nepal [14], Mexico [15], Chile [16] and Hungary [17]. There is no Arabic form of the EPDS that has been validated. However, a study conducted on a sample of 228 Lebanese women concluded that EPDS may be regarded as a reliable screening tool as early as 30–40 days after delivery [18].

Perinatal anxiety is another vulnerability that women are prone to develop during pregnancy and after giving birth. Anxiety is experienced throughout pregnancy (antenatal) and/or the postpartum period (12 months after birth) [19]. Perinatal anxiety has been recognized as a reliable leading indicator of postpartum depression [20]. A study carried out by Miller et al. detected that depression can be the consequence of undiagnosed and untreated anxiety [21]. Another study found that women with an anxiety disorder during pregnancy are at three times greater risk of developing postpartum depression [20].

To screen for perinatal anxiety, the Perinatal Anxiety Screening Scale (PASS) is adopted for a wide range of anxiety disorders, as well as some common perinatal-specific fears. This scale was also validated in other languages [22,23] but not Arabic. It is a valid and useful tool for identifying pregnant women and new mothers with problematic anxiety.

Limited research is available on the assessment of risk factors for postpartum depression and postpartum anxiety. Clout and Brown reported that several factors associated with postpartum depression have a limited correlation to postpartum anxiety (e.g., education, income and breastfeeding difficulties) [24]. Recent studies confirmed that factors for postpartum anxiety include unplanned pregnancy, lack of control during labor, pain, postpartum depression, poor physical health and low partner support [25,26].

At a local level, a 2020 systematic literature review was the largest in the Arab region [27]. Based on 25 studies, it reviewed risk factors and the prevalence of postpartum depression among Arab mothers. Compared with the Western region, higher postpartum depression prevalence was found in the Arab region [27]. Postpartum depression is considered high among Arab mothers and is projected to affect around 1 in 5 mothers [27].

In Lebanon, scarce data on postpartum depression are retrievable. Three research studies aimed at assessing mothers for postpartum depression. Chaaya et al. [28] and El-Hachem et al. [18] worked on evaluating the prevalence and determinants, as well as identifying women at risk of postpartum depression respectively. The third study by Awad et al. (2020) was a national study and more general; it studied pregnant women’s knowledge, perceptions and fears, including postpartum depression [29]. The overall prevalence of postpartum depression in the Lebanese population was 21.3% in one study [28]. In the second study, the prevalence of postpartum depression was 12.8% between days 30 and 40 [18].

Socially, Lebanese citizens are at an increased risk of being unemployed, losing their job and having their salaries cut [29]. These hurdles set the tone for the kind of pressure the Lebanese population, especially mothers, are up against on a daily basis. Hence, it is important to screen for socio-demographic factors among the risk factors for postpartum depression. The Lebanese population comprises a set of factors that play a role in the depression and anxiety of mothers that are categorically different when compared with the general literature.

The last study conducted in Lebanon about postpartum depression dates back to 2014 [30]; it is the only previous research study that assessed the reliability of EPDS as an early screening tool for postpartum depression. Little information is known about the factors that influence the prevalence of postpartum depression among Lebanese women. Moreover, no previous studies were conducted to assess postpartum anxiety. Postpartum morbidities in Lebanon were never previously thoroughly investigated. The shortage of research in this field and the potential opportunities to provide different aspects of postpartum care that respond to women’s needs require a deeper understanding of the various problems faced by postpartum women.

This study’s main objective was to delineate the factors associated with postnatal depression (i.e., postpartum anxiety, insomnia, factors related to newborn, delivery complications, lack of support and a delivery period of 14 h) and postnatal anxiety (i.e., postpartum depression, use of technology help for delivery, premature baby birth, high perceived stress and adverse life events), as well as to linguistically validate the EPDS and PASS scales in the Arabic language, among a sample of Lebanese women 4–6 weeks after delivery. We hypothesized that the psychological factors (history of depression, higher insomnia, higher perinatal anxiety), psychosocial factors (marital problems, decreased social support, higher stress), socio-economic factors (unemployment, poverty, low education level), medical factors (complications during delivery and pregnancy) and factors related to the child (girl baby, difficulties in breastfeeding, undesired pregnancy) would be associated with higher postpartum depression.

## 2. Materials and Methods

### 2.1. Study Design

This cross-sectional study was carried out between July 2018 and March 2019. Participants were recruited from four gynecologists’ clinics located in four different districts in Lebanon. Data collection was carried out at each of these gynecologist clinics using study-independent personnel considered as assessors. For the choice of participants, each Lebanese married woman aged more than 18 years old who came for a postnatal checkup to the clinic 4–6 weeks after delivery was asked if she would like to participate in this study, and after her written consent was given, she was considered as a participant. All types of pregnancies were accepted in this study, whether high-risk or minimal-risk pregnancies. Exclusion criteria: patients excluded were women with a physician-diagnosed mental illness (previously diagnosed psychiatric disorders), women with intellectual disabilities or women who refused to take part in the study. None of the participants dropped out of the study.

### 2.2. Sample Size Calculation

The Epi info program was used to calculate the minimum sample size needed for our study with an acceptable 5% margin of error, an expected 12.5% frequency of women with postpartum depression [6] and an estimated 86,000 births per year in Lebanon [31], the results indicated that we needed 168 participants to participate in the study.

### 2.3. Survey Details

The survey was administered at the obstetricians’ clinics in Arabic without the presence of a third-party observant (husband, family member, etc.) and took approximately 25 min to be completed. The questionnaire was self-administered to the mother unless she was illiterate, in which case the investigator helped out by reading the questions for her. The first part of the questionnaire consisted of the sociodemographic features, including age, gender, region, level of education, professional status, insurance type, history of diseases, and other questions that were linked to factors associated with postpartum depression and anxiety according to the literature review [9,11,12,32].

The second part consisted of the scales used in the study as follows:

- We used the Edinburgh Postnatal Depression Scale (EPDS) to screen for the possible presence of postpartum depression. It is the standardized tool used postnatally to quantify the severity and establish an estimation of postpartum depression [33]. EPDS is a valid 10-question scale that is valuable for identifying the potential risk of depression following childbirth and an effective screening tool that demonstrates sensitivity and high reliability. Regarding the scoring of the EDPS questionnaire, the following is used: answers are scored on a scale from 0 (not at all) to 3 (as much as I ever did). Therefore, the total score ranges from 0 to 30, with a score of 11 or more deemed positive for postpartum depression. On the total EPDS score, the threshold value ≥11 is deemed a relevant diagnostic criterion in order to diagnose appropriately postnatal depression during the post-delivery time range of 4–6 weeks [33].
- The Lebanese Insomnia Scale (LIS), which is a validated scale for assessing insomnia in Lebanon [34], is composed of 18 items used to screen for the diagnosis of insomnia based on several validated and universally applicable self-report scales. It aims to provide a valid, standardized and reliable reflection of sleep quality. Answers are scored according to a scale (1 = never and 5 = always); greater scores designate worse insomnia. Cronbach’s alpha value for this scale was 0.732.
- We also included the “Presumptive Stressful Life Events Scale” (PSLES) to evaluate whether there was one of several stressful life events that may have happened to the mother up to 12 months before giving birth to her newborn. This scale was constructed and standardized for two periods: the previous one year and over her lifetime. The scale events are divided into 9 categories: family and social, work, financial, marital and sexual, health, bereavement, education, legal and, finally, courtship and cohabitation. Each category has a series of events, where the mother should respond with a “yes” if the event occurred during the last year or/and during her lifetime. Cronbach’s alpha value for this scale was excellent (0.967).
- This questionnaire also contained the “Perinatal Anxiety Screening Scale” (PASS) to detect the severity of perinatal anxiety. The PASS is a trustworthy 31-item self-reported questionnaire for postpartum and antenatal women to screen for anxiety. It distinguishes between high and low anxiety disorder risk by measuring specific anxiety symptoms. The mother scores these symptoms by indicating their frequency over the last month. The scale is as follows: 0 (not at all), 1 (sometimes), 2 (often) and 3 (always). Scores between 0 and 20 indicate the absence of anxiety symptoms, scores between 21 and 41 indicate mild-to-moderate symptoms, and scores between 42 and 93 indicate severe symptoms. High and low anxiety disorder risks were separated with a cut-off score of 26.

### 2.4. Forward and Back Translation

A protocol of initial translation followed by a back translation was adopted to translate all English scales used to Arabic. The initial translation from English to Arabic was carried out by a mental health professional, after which a different specialist translated this version in Arabic back again to English. To ensure the reliability of both translations at the end of the translation protocol, both English versions were compared. This comparison yielded no significant difference between the versions.

### 2.5. Statistical Analysis

The FACTOR program was used to conduct the exploratory factor analyses of the depression and anxiety questionnaire in the Lebanese population by using the Pearson correlation technique, a parallel analysis to determine the number of factors and a promax rotation since items were found to be significantly associated. The Kaiser–Meyer–Olkin (KMO) measure of sampling adequacy and Bartlett’s test of sphericity were applied and found to be adequate. The retained factors matched were those with eigenvalues higher than one. Moreover, Cronbach’s alpha was recorded for the reliability analysis for the total score and subscale factors. Statistical analysis was carried out using SPSS software version 23. The normality of the distribution of the postpartum depression and anxiety scores was confirmed via a calculation of the skewness and kurtosis; values for asymmetry and kurtosis between −1 and +1 are considered acceptable in order to demonstrate a normal univariate distribution [35]. Student’s t and ANOVA tests were used to observe differences between the means of two and three or more groups, respectively. The Bonferroni-corrected *p*-value was calculated for multiple testing by dividing 0.05 by the number of factors tested (=33); in the bivariate analysis, significance was assumed at *p* = 0.05/33 = 0.001. Concerning the multivariable analyses, four stepwise linear regressions were conducted; the first two were conducted by taking the postpartum depression and anxiety scores as dependent variables but without taking them as independent variables in the models, whereas the other two models included one each of these variables as independent variables. Significance was defined as *p* < 0.05 in the final models.

## 3. Results

### 3.1. Sociodemographic Characteristics of the Participants

Out of 350 women approached, 295 (84.28%) accepted the offer to participate in this study. The results of the sociodemographic characteristics of the participants are summarized in Table 1. The results showed that the mean age of the mothers at delivery was 29.53 ± 5.18 years, with 75.6% having a university level of education, 50.7% having a monthly income between USD 1000–2000 and 40.8% having a national social security funds (NSSF) insurance type. Moreover, 112 (38.2%) of the mothers had no depression, 44 (15.0%) had possible depression, 35 (11.9%) had a fairly high possibility of depression and 102 (34.8%) had a positive screening for depression. Finally, 92 (32.3%) of the mothers had no anxiety symptoms, 156 (54.7%) had mild-to-moderate symptoms and 37 (13.0%) had severe symptoms.

### 3.2. Principal Component Analysis

None of the items of the EPDS and PASS scales was removed. The EPDS and PASS scales’ items converged over two- and four-factor solutions, explaining 62.51% and 53.33% of the variance, respectively (KMO EPDS = 0.816, αCronbach EPDS = 0.826; KMO PASS = 0.878, αCronbach PASS = 0.920; Bartlett’s test of sphericity *p* < 0.001) (Table 2). In terms of discriminant validity, no ceiling or floor effects were detected (Table 3).

### 3.3. Bivariate Analysis

#### 3.3.1. Bivariate Analysis Associated with Postpartum Depression and Anxiety

The results of the bivariate analyses of factors associated with postpartum depression and anxiety are summarized in Table 4 and Table 5. A higher mean depression score was found in mothers who were delivering their third child or more (12.84), in those whose babies woke up more than five times at night (14.04) and in those whose babies did not eat regularly compared with those who did (13.47 vs. 10.22). Moreover, significantly higher mean depression scores were found in mothers who had hypotension during pregnancy (15.33 vs. 10.55) and in those who had higher postpartum anxiety (r = 0.628) and insomnia (r = 0.359).

#### 3.3.2. Bivariate Analysis Associated with Postpartum Anxiety

A higher mean anxiety score was found in mothers whose babies woke up more than five times during the night and who did not eat regularly compared with those who did (32.76 vs. 26.33). Moreover, significantly higher mean anxiety scores were found in mothers who did not have planned pregnancies (30.76 vs. 25.68), in those who were not happily married (35.38 vs. 27.01) and in those who had a delivery time of 20 h or more (35.86). Finally, higher insomnia (r = 0.323) was found to be significantly correlated with higher postpartum anxiety.

### 3.4. Multivariable Analysis

The results of a first stepwise linear regression, taking the postpartum depression score as the dependent variable and without taking the postpartum anxiety score as an independent variable, showed that higher postpartum insomnia (Beta = 0.173), having hypotension during pregnancy (Beta = 3.680), having a third or more baby compared with the first one (Beta = 1.975) and having a baby who woke up more than 5 times per night compared with 1–2 times (Beta = 1.942) were significantly associated with higher postpartum depression (Table 6, model 1). The results of a second stepwise linear regression, taking the postpartum anxiety score as the dependent variable and without taking the postpartum depression score as an independent variable, showed that higher postpartum insomnia (Beta = 0.290) and having a baby who woke up more than 5 times per night compared with 1–2 times (Beta = 4.295) were significantly associated with higher postpartum anxiety, whereas having a planned pregnancy compared with not (Beta = −5.174) and the fact that the baby ate regularly compared with not (Beta = −4.657) were significantly associated with lower postpartum anxiety (Table 6, model 2). The results of a third stepwise linear regression, taking the postpartum depression score as the dependent variable and taking the postpartum anxiety score as an independent variable, showed that higher postpartum anxiety (Beta = 0.256), higher postpartum insomnia (Beta = 0.079), having hypotension during pregnancy (Beta = 2.760), and having a second (Beta = 1.663) or a third or more baby (Beta = 2.470) compared with the first one were significantly associated with higher postpartum depression (Table 6, model 3).

The results of a fourth stepwise linear regression, taking the postpartum anxiety score as the dependent variable and taking the postpartum depression score as an independent variable, showed that higher postpartum depression (Beta = 1.33) was significantly associated with higher postpartum anxiety, whereas having a baby through a planned pregnancy (Beta = −4.365) and having a baby who ate regularly (Beta = −3.639) were significantly associated with lower postpartum anxiety (Table 6, model 4).

## 4. Discussion

The current study identified the clinical factors associated with postpartum depression and anxiety among a sample of Lebanese women. In this study, the preliminary psychometric properties of the EDPS and PASS scales were satisfactory, showing that these two scales were adequate to screen for postpartum depression and anxiety in Lebanese women while identifying some factors associated with them.

### 4.1. Validation of Both Scales:

In our study, the EPDS scale items converged over a two-factor solution that outlined a total of 62.51% of the variance, with an internal consistency of 0.826. Several validation studies in different countries confirmed the clinical and epidemiological value of the scale: in Chile, the items of the EPDS converged over one factor with a Cronbach’s alpha of 0.914 [16], while in France, the EPDS items converged over a solution of two factors, with a Cronbach’s alpha of 0.76 [36]. Our results confirmed that the Arabic version of the instrument had good preliminary psychometric properties. The research conducted by El Hachem et al. in 2014 validated the use of EPDS as a trusted indicator for the risk of developing postpartum depression [18].

As for the PASS scale items, in our study, it joined over a total of four factors, explaining a total of 53.33% of the variance and leading to an internal consistency of 0.920. These findings were similar to those of the original developers in terms of the number of factors and internal consistency (Cronbach’s alpha = 0.96) [37]. The validation of this scale in Turkey [23] revealed that the Cronbach’s alpha value for the scale was  0.95, and the sub-dimensions obtained using explanatory factor analysis were (1) general anxiety and specific fear, (2) perfectionism and control, (3) social anxiety and adjustment disorder, and (4) acute anxiety and trauma. This test was also validated in Bangladesh [22] and the exploratory factor analysis showed a four-factor solution of the Bangla PASS: (1) acute anxiety, (2) general worry and specific fears, (3) perfectionism control and trauma, and (4) social anxiety; meanwhile, the temporal stability and internal consistency were also satisfactory (Cronbach’s alpha = 0.970). The Australian validation, in turn, suggested a four-factor structure addressing symptoms of (1) acute anxiety and adjustment; (2) general worry and specific fears; (3) perfectionism, control and trauma; and (4) social anxiety, with excellent reliability (Cronbach’s α = 0.96) [37].

Accordingly, the preliminary results suggested that these two scales are adequate for the assessment of postpartum depression and anxiety among Lebanese women. This study revealed that the rate of depression and anxiety in the postpartum Lebanese population was 61.8% and 67.7%, respectively, higher than those of a previous study (occurrence of depression was 5.1% at the fourth week of postpartum and 5.7% at 2 months postpartum) [38]. In addition, it was reported that the prevalence of postpartum depression affects about 10–15% of adult mothers annually, with depressive symptoms lasting more than 6 months [39]. The rate of depression in this study was similar to another study conducted by Halbreich et al. that reported an approximately 60% prevalence rate of depression [40].

As for anxiety prevalence, the range was reported to be from 13 to 40%, which was lower than the findings in this study [25]. This wide variation in the anxiety prevalence rates is highly reliant on the type of anxiety assessment, the scale depicted in the assessment, the cut-off score for anxiety, the severity of anxiety, the timing and the frequency of the assessment, and the country of origin [25,41]. In this study, the high rates of anxiety in the postpartum period may relate to the fact that the anxiety scale scores were based on interviews where denying symptoms may be hindered through face-to-face contact.

### 4.2. Factors Associated with Depression

In this study, the factors that were associated with postpartum depression were higher postpartum anxiety, insomnia and complications development during pregnancy. Furthermore, an increased number of awakenings of the baby during the night was significantly associated with higher depression. In this study, insomnia was highly associated with the development of postpartum depression since sleep deprivation might be a trigger factor for the onset of certain psychological problems encountered post-delivery, as the onset of mania and an unbalanced sleep pattern are more prevalent in new mothers [42]. The maintenance of balanced sleeping hours aids in relaxation and minimizes the risk of depression.

Our study found that complications during pregnancy (hypotension) were associated with postpartum depression, which can be explained by the emergence of physical and mental troubles encountered by the mother due to the fear of the consequences of complications development; this outcome is consistent with the outcomes of other studies [43,44].

### 4.3. Factors Associated with Anxiety

Previous findings observed the combined relationship between symptoms of anxiety and postpartum depression [27]. The onset of anxiety can range from a few days to a few weeks after delivery and usually peaks in the first 2–3 months following childbirth. Postpartum anxiety appears mainly in mothers who have a fear of cot death. One plausible explanation that might elaborate on the reason why this accumulation of fear over time leads to postpartum anxiety is that nocturnal vigilance deprives mothers of a normal sleeping pattern since this causes them to remain awake listening to the breathing of the infant. Therefore, the irregular sleeping pattern and anxiety (through constant worrying) seem to be related. This recurrent checking on the safety and health of their children predisposes the mother to anxiety and depression [45].

In our study, anxiety was associated with planned pregnancy and an indifferent attitude to pregnancy. An unwanted pregnancy may significantly change one’s life; be a stressful experience with different impacts on one’s quality of life; and may trigger certain psychological problems, such as anxiety [46]. In our study, the delivery of a premature baby was associated with postpartum anxiety, in agreement with previous findings [47]. The underlying reasons for this relationship can be depicted by the isolation that the parents are exposed to after the delivery of premature babies, where hospitalization is mandated due to the difficulty in discharging premature infants without being admitted to neonatal intensive care units. The isolation of infants and a lengthy hospital stay pose sudden changes in the bonding of the parents with their offspring [48]. The early relationship between parents and newborn infants encountered in the first moments immediately post-delivery is fundamental and plays a crucial role in this intimate bonding [49]. During the hospital stay of premature babies, mothers often experience negative thoughts and ideas and contradictory emotional reactions that are usually diagnosed as grief, sorrow, guilt, fear, anger, loss of self-esteem and a sense of failure [47]. This situation and these feelings predispose mothers to anxiety. The factors associated with postpartum depression and anxiety were extensively studied throughout different cultures and countries. The conclusions drawn from this study affect current knowledge in the field by adding a localized approach to the studied factors and validation of EPDS and PASS scales. The results of our study generally offer validation of the factors when compared with the existing literature. The postpartum anxiety and depression results reported in the literature are lower than those found in our study. We hypothesize that this offers insight into the seriousness of postpartum morbidity in Lebanon. Future research can build on these observations by evaluating individual factors and factors associated with both postpartum depression and postpartum anxiety in various local communities in order to draw comparisons. In Lebanon, a larger national study can be established so that the preliminary validation of EPDS and PASS scales can be fully validated. We suggest designing key studies using more advanced psychometric tools. The optimal design will be achieved via coordination between psychologists/psychiatrists and obstetricians since they deal with postpartum morbidities on the front line.

The results from this study can be extrapolated to the Lebanese sample of women. The factors delineated the main problems faced by postpartum women. By knowing exactly which factors affect postpartum depression and postpartum anxiety, awareness can be raised among pregnant females so that they are offered counseling options that help them to cope with this psycho-physiological stress on their bodies. In a more general aspect, postpartum anxiety was found to be a leading indicator that directly affects postpartum depression [50]. We postulate that the proper screening and management of anxiety may have a negative retrocontrolling effect on depression.

### 4.4. Implications for Psychiatric Practice

The proper assessment of factors associated with postpartum depression and postpartum anxiety among Lebanese women allows for appropriate action plans and targeted treatments. This highly prevalent problem of postpartum depression and anxiety among Lebanese women has several risk factors. An interplay of these factors is likely to play a role in causing postpartum depression and anxiety. Taking care of these highly modifiable risk factors can prevent postpartum depression and postpartum anxiety development. Thus, early recognition of risk factors for postpartum depression/postpartum anxiety may aid clinicians in early intervention and management. A collaborative care approach would be appropriate to identify high-risk mothers for postpartum depression and postpartum anxiety development. Resolving marital and family conflicts before conception, helping the mothers to draw a support plan, having realistic expectations of birth and parenting, addressing issues of self-esteem, and encouraging them to quit smoking and waterpipes might be some of the ways to prevent postpartum disorders. We also recommend that a psychiatrist and a psychologist attend a postnatal care unit to advise mothers at risk of developing postpartum depression or postpartum anxiety, as well as other psychiatric disorders.

### 4.5. Limitations

There are some limitations to this study. The prevalence rates of perinatal depression were assessed in this study using self-reported instruments, such as EPDS, which are not considered solid evidence regarding the clinical depression diagnosis and typically overestimate incidence rates [51,52]. The EPDS is a screening test that requires further diagnostic confirmation through a structured or semi-structured interview. Consequently, accurate conclusions cannot be drawn. The utilization of a comprehensive tool, such as the Patient Health Questionnaire, that aids in screening, diagnosing, monitoring and measuring the intensity of depression is a more useful instrument. In addition, there might have been an information bias where participants might either over- or underestimate their symptoms. Furthermore, a selection bias might be present since the sample was taken from doctor’s clinics and is not representative of the whole population.

## 5. Conclusions

Depression and anxiety rates in Lebanese postpartum women seemed to be higher compared with other countries, which may in part be due to differences in regional, social, and environmental culture. It is worth concluding that great importance should be given by healthcare professionals to implementing policies that raise awareness about postpartum depression and create health promotion programs to increase the well-being of birthing women. Different etiological factors could contribute to both depression and anxiety in the postpartum period, which could adversely affect both the mother and the infant. Preventive techniques should be employed early before delivery and even continued post-delivery to aid mothers in the most effective ways of how to cope with the situations and feelings experienced in this period.

## Figures and Tables

**Table 1:** Sociodemographic characteristics of the participants.

Variable	N (%)
**Sex of the baby** Male Female	152 (51.7%) 142 (48.3%)
**Mother’s educational level** Illiterate/primary Complementary Secondary University	18 (6.1%) 14 (4.7%) 40 (13.6%) 223 (75.6%)
**Monthly income**	
No income <USD 1000 USD 1000–2000 >USD 2000	53 (18.8%) 42 (14.9%) 143 (50.7%) 44 (15.6%)
**Insurance type** No insurance Private insurance NSSF Private and NSSF Army COOP	29 (10.2%) 56 (19.7%) 116 (40.8%) 49 (17.3%) 22 (7.7%) 12 (4.2%)
**Governorate**	
Beirut	32 (10.9%)
Mount Lebanon	104 (35.5%)
North	44 (15.0%)
South	109 (37.2%)
Bekaa	4 (1.4%)
	**Mean ± SD**
**Mother’s age at delivery (years)**	29.53 ± 5.18
**Father’s age at delivery (years)**	34.32 ± 5.87
**Postpartum depression**	10.90 ± 6.17
**Postpartum anxiety** **Postpartum insomnia** **Stress: previous year score** **Stress: lifetime score**	27.76 ± 13.23 47.88 ± 10.11 5.22 ± 8.06 2.86 ± 5.21

**Table 2:** Factor analyses using the promax rotation.

Model 1: Factor Analysis of the Edinburgh Postnatal Depression Scale (EPDS)					
Factor	Items	Factor 1	Factor 2	Factor 3	Factor 4
I have been able to laugh and see the funny side of things	EPDS 1		0.869		
I have looked forward with enjoyment to things	EPDS 2		0.795		
I have blamed myself unnecessarily when things went wrong	EPDS 3	0.896			
I have been anxious or worried for no good reason	EPDS 4		0.780		
I have felt scared or panicky for no very good reason	EPDS 5	0.708			
Things have been getting on top of me	EPDS 6	0.884			
I have been so unhappy that I have had difficulty sleeping	EPDS 7	0.729			
I have felt sad or miserable	EPDS 8	0.735			
I have been so unhappy that I have been crying.	EPDS 9	0.759			
The thought of harming myself has occurred to me	EPDS 10		0.467		
Cronbach’s alpha					
Percentage of variances explained		48.84	13.67		
**Model 2: Factor Analysis of the Perinatal Anxiety Screening Scale (PASS)**					
**Factor**	**Items**	**Factor 1**	**Factor 2**	**Factor 3**	**Factor 4**
Worry about the baby/pregnancy	PASS 1			0.922	
Fear that harm will come to the baby	PASS 2			0.880	
A sense of dread that something bad is going to happen	PASS 3			0.661	
Worry about many things	PASS 4			0.768	
Worry about the future	PASS 5			0.489	
Feeling overwhelmed	PASS 6	0.587			
Really strong fears about thing (needles, blood, birth, pain, etc)	PASS 7			0.431	
Sudden rushes of extreme fear or discomfort	PASS 8	0.607			
Repetitive thoughts that are difficult to stop or control	PASS 9	0.496			
Difficulty sleeping even when I have the chance to sleep	PASS 10			0.300	
Having to do things in a certain way or order	PASS 11				0.714
Wanting things to be perfect	PASS 12				0.887
Needing to be in control of things	PASS 13				0.787
Difficulty stopping checking or doing things over and over	PASS 14	0.790			
Feeling jumpy or easily startled	PASS 15	0.631			
Concerns about repeated thoughts	PASS 16	0.516			
Being ‘on guard’ or needing to watch out for things	PASS 17				0.471
Upset about repeated memories, dreams or nightmares	PASS 18	0.722			
Worry that I will embarrass myself in front of others	PASS 19	0.804			
Fear that others will judge me negatively	PASS 20	0.498			
Feeling really uneasy in crowds	PASS 21		0.743		
Avoiding social activities because I might be nervous	PASS 22		0.795		
Avoiding things which concern me	PASS 23				0.540
Feeling detached like you’re watching yourself in a movie	PASS 24			0.779	
Losing track of time and can’t remember what happened	PASS 25			0.677	
Difficulty adjusting to recent changes	PASS 26			0.786	
Anxiety getting in the way of being able to do things	PASS 27			0.712	
Racing thoughts making it hard to concentrate	PASS 28			0.547	
Fear of losing control	PASS 29			0.452	
Feeling panicky	PASS 30			0.636	
Feeling agitated	PASS 31			0.711	
Cronbach’s alpha					
Percentage of variances explained		32.22	9.57	6.58	4.96

**Table 3:** Frequency of participants according to the depression and anxiety scores.

	Depression	Anxiety
0	6 (2.0%)	1 (0.4%)
1	8 (2.7%)	1 (0.4%)
2	13 (4.4%)	0 (0%)
3	13 (4.4%)	1 (0.4%)
4	14 (4.8%)	1 (0.4%)
5	19 (6.5%)	2 (0.7%)
6	12 (4.1%)	0 (0%)
7	12 (4.1%)	8 (2.8%)
8	15 (5.1%)	7 (2.5%)
9	14 (4.8%)	5 (1.8%)
10	16 (5.5%)	3 (1.1%)
11	14 (4.8%)	4 (1.4%)
12	16 (5.5%)	3 (1.1%)
13	19 (6.5%)	3 (1.1%)
14	13 (4.4%)	5 (1.8%)
15	12 (4.1%)	13 (4.6%)
16	16 (5.5%)	4 (1.4%)
17	10 (3.4%)	8 (2.8%)
18	15 (5.1%)	8 (2.8%)
19	9 (3.1%)	4 (1.4%)
20	12 (4.1%)	11 (3.9%)
21	2 (0.7%)	6 (2.1%)
22	5 (1.7%)	5 (1.8%)
23	4 (1.4%)	9 (3.2%)
24	2 (0.7%)	10 (3.5%)
25	1 (0.3%)	2 (0.7%)
26	0 (0%)	4 (1.4%)
27	0 (0%)	6 (2.1%)
28	1 (0.3%)	14 (4.9%)
29		2 (0.7%)
30		4 (1.4%)
31		13 (4.6%)
32		13 (4.6%)
33		9 (3.2%)
34		9 (3.2%)
35		8 (2.8%)
36		13 (4.6%)
37		4 (1.4%)
38		5 (1.8%)
39		7 (2.5%)
41		13 (4.6%)
42		9 (3.2%)
43		4 (1.4%)
44		6 (2.1%)
46		4 (1.4%)
48		1 (0.4%)
49		2 (0.7%)
51		1 (0.4%)
54		2 (0.7%)
56		1 (0.4%)
57		1 (0.4%)
59		1 (0.4%)
67		2 (0.7%)
71		2 (0.7%)
81		1 (0.4%)

**Table 4:** Bivariate analysis of categorical variables associated with postpartum depression and anxiety among the participants.

Variable	Depression Score	*p*-Value	Anxiety Score	*p*-Value
**Governorate**		0.374		0.003
Beirut	11.53 ± 6.77		31.44 ± 13.87	
Mount Lebanon	10.16 ± 6.55		24.54 ± 14.19	
North	11.07 ± 5.55		26.09 ± 12.90	
South	11.49 ± 5.77		30.85 ± 11.38	
Bekaa	7.25 ± 7.22		33.25 ± 5.25	
**Mother’s education level**		0.014		0.048
Illiterate/primary	13.55 ± 3.07		34.65 ± 4.86	
Complementary	13.85 ± 6.02		26.85 ± 10.70	
Secondary	9.17 ± 6.18		27.72 ± 14.40	
University	10.80 ± 6.26		27.43 ± 13.52	
**Insurance type**		0.073		0.859
No insurance	8.76 ± 5.77		26.28 ± 13.17	
Private insurance	9.80 ± 5.53		27.19 ± 15.06	
NSSF	12.08 ± 5.95		27.75 ± 11.49	
Private and NSSF	11.00 ± 7.44		28.91 ± 15.55	
Army	9.86 ± 6.24		29.41 ± 12.79	
COOP	10.58 ± 5.55		31.45 ± 13.27	
**Number of deliveries**		**<0.001**		0.192
First child	9.29 ± 6.25		26.52 ± 13.84	
Second child	11.47 ± 5.48		27.96 ± 12.42	
Third child or more	12.84 ± 6.21		30.02 ± 12.94	
**Gestational week**		0.152		0.057
Premature birth	11.48 ± 7.08		32.50 ± 14.41	
At term but weighed less than normal	9.10 ± 5.37		26.11 ± 11.29	
At term with normal weight	11.04 ± 6.16		27.02 ± 13.37	
**Satisfied with the sex of the baby**		0.01		0.153
No	13.77 ± 8.18		35.89 ± 17.60	
Yes	10.72 ± 6.07		27.49 ± 13.14	
Do not know	18.00 ± 3.39		32.80 ± 2.95	
**Delivery mode**		0.252		0.006
Normal delivery	10.02 ± 5.64		24.48 ± 11.68	
Normal delivery with help during delivery	11.05 ± 5.16		28.68 ± 14.36	
Cesarian section	11.31 ± 6.55		29.81 ± 13.61	
**Sex of the baby**		0.102		0.02
Male	11.44 ± 6.44		29.64 ± 13.53	
Female	10.26 ± 5.82		26.01 ± 12.67	
**Admission to NICU**		0.057		0.004
No	10.56 ± 6.06		26.77 ± 13.36	
Yes	12.37 ± 6.59		32.63 ± 11.68	
**Baby woke up at night**		**<0.001**		**<0.001**
1–2 times	9.17 ± 6.14		24.08 ± 14.52	
3–5 times	10.95 ± 5.85		28.46 ± 11.68	
>5 times	14.04 ± 5.56		34.43 ± 12.05	
**Baby eats regularly**		**<0.001**		**0.001**
No	13.47 ± 6.12		32.76 ± 11.94	
Yes	10.22 ± 5.99		26.33 ± 13.52	
**Baby had health problems**		0.013		0.029
No	10.43 ± 5.86		27.05 ± 12.34	
Yes	14.13 ± 7.44		35.14 ± 18.57	
**Complications during pregnancy**		0.005		0.003
No	10.19 ± 6.11		26.21 ± 12.46	
Yes	12.44 ± 6.11		31.36 ± 14.44	
**Hypotension during pregnancy**		**<0.001**		0.029
No	10.55 ± 6.21		27.27 ± 13.10	
Yes	15.33 ± 3.77		34.33 ± 14.26	
**Loss of consciousness during pregnancy**		0.018		0.074
No	10.71 ± 6.17		27.50 ± 13.31	
Yes	16.28 ± 4.50		36.57 ± 7.81	
**Hospitalized during pregnancy**		0.022		0.005
No	10.52 ± 6.01		26.87 ± 12.45	
Yes	12.64 ± 6.64		32.55 ± 15.39	
**Complications during delivery**		0.016		0.102
No	10.47 ± 6.12		27.22 ± 13.41	
Yes	12.81 ± 6.17		30.68 ± 12.04	
**Anemia after delivery**		0.013		0.09
No	10.58 ± 6.14		27.54 ± 12.97	
Yes	13.91 ± 5.80		32.55 ± 16.49	
**Technological help during delivery**		0.236		0.027
No	11.08 ± 6.20		27.61 ± 13.56	
Yes	9.19 ± 6.05		32.71 ± 7.27	
**Previous abortions**		0.037		0.164
No	10.49 ± 6.03		27.27 ± 12.49	
Yes	12.28 ± 6.50		19.86 ± 15.35	
**Planned pregnancy**		0.006		**0.001**
No	12.07 ± 6.37		30.76 ± 12.73	
Yes	10.07 ± 5.89		25.68 ± 13.22	
**Happy with married life**		0.008		**0.001**
No	13.65 ± 5.83		35.38 ± 13.63	
Yes	10.51 ± 6.08		27.01 ± 12.98	
**Alcohol during pregnancy**		0.065		0.106
No	10.90 ± 6.14		27.36 ± 13.38	
Yes	13.93 ± 6.65		33.07 ± 10.65	
**Delivery period**		0.06		**<0.001**
14 h	8.90 ± 4.74		18.54 ± 10.07	
20 h or more	13.40 ± 4.34		35.86 ± 9.63	
Less than 14 h	9.75 ± 6.45		26.57 ± 14.08	

**Table 5:** Pearson’s correlations of continuous variables associated with postpartum depression and anxiety among the participants.

Variable	Depression		Anxiety	
	r	*p*-Value	r	*p*-Value
Depression	1	-	0.628	**<0.001**
Anxiety	0.628	**<0.001**	1	-
Insomnia	0.359	**<0.001**	0.323	**<0.001**
Stress during previous year	0.037	0.532	0.058	0.331
Stress during lifetime	0.028	0.636	0.013	0.833
Mother’s age at delivery	−0.09	0.124	−0.086	0.148
Father’s age at delivery	−0.06	0.307	−0.033	0.585
Number of previous abortions	0.184	0.155	0.057	0.664
Number of cigarettes per day	−0.076	0.845	0.595	0.091
Number of waterpipes per week	0.168	0.549	0.590	0.021

Numbers in bold indicate significant *p* values.

**Table 6:** Multivariable analysis.

Model 1: Stepwise Linear Regression Taking the Postpartum Depression Score as the Dependent Variable without Taking the Postpartum Anxiety Score as an Independent Variable				
Variable	Unstandardized Beta	*p*-Value	95% Confidence Interval	
Postpartum insomnia score	0.173	<0.001	0.104	0.242
Hypotension during pregnancy (yes vs. no *)	3.68	0.008	0.953	6.407
Third or more child vs. first *	1.975	0.014	0.404	3.546
Baby woke up more than 5 times during the night vs. 1–2 times *	1.942	0.035	0.137	3.747
* Reference group; Nagelkerke R^2^ for model 1 = 18.3%				
**Model 2: Stepwise Linear Regression Taking the Postpartum Anxiety Score as the Dependent Variable without Taking the Postpartum Depression Score as an Independent Variable**				
**Variable**	**Unstandardized Beta**	***p*-Value**	**95% Confidence Interval**	
Insomnia score	0.290	<0.001	0.131	0.449
Planned pregnancies (yes vs. no *)	−5.174	0.002	−8.364	−1.984
Baby ate regularly (yes vs. no *)	−4.657	0.012	−8.273	−1.041
Baby woke up more than 5 times per night compared with 1–2 times *	4.295	0.033	0.341	8.250
* Reference group; Nagelkerke R^2^ for model 2 = 18.0%				
**Model 3: Stepwise Linear Regression Taking the Postpartum Depression Score as the Dependent Variable and Taking the Postpartum Anxiety Score as an Independent Variable**				
**Variable**	**Unstandardized Beta**	***p*-Value**	**95% Confidence Interval**	
Postpartum anxiety score	0.256	<0.001	0.211	0.301
Postpartum insomnia score	0.079	0.009	0.020	0.139
Third or more child vs. first *	2.470	0.001	1.052	3.888
Hypotension during pregnancy (yes vs. no *)	2.760	0.014	0.555	4.964
Second child vs. first *	1.663	0.017	0.304	3.023
* Reference group; Nagelkerke R^2^ for model 3 = 46.6%				
**Model 4: Stepwise Linear Regression Taking the Postpartum Anxiety Score as the Dependent Variable and Taking the Postpartum Depression Score as an Independent Variable**				
**Variable**	**Unstandardized Beta**	***p*-Value**	**95% Confidence Interval**	
Postpartum depression score	1.33	<0.001	1.116	1.543
Planned pregnancy (yes vs. no *)	−4.365	0.001	−6.943	−1.787
Baby ate regularly (yes vs. no *)	−3.639	0.015	−6.564	−0.713

* Reference group; Nagelkerke R^2^ for model 4 = 44.7%.

## Data Availability

Not applicable.
